# Determining the optimal 5-FU therapeutic dosage in the treatment of colorectal cancer patients

**DOI:** 10.18632/oncotarget.11980

**Published:** 2016-09-12

**Authors:** Ling Fang, Yi Jiang, Yuxian Yang, Yuqiong Zheng, Jin Zheng, Hong Jiang, Shengqi Zhang, Lifang Lin, Jieting Zheng, Shuyao Zhang, Xiaowen Zhuang

**Affiliations:** ^1^ Pharmacy Intravenous Admixture Service, Cancer Hospital of Shantou University Medical College, Shantou, Guangdong, China; ^2^ Digestive Medical Oncology, Cancer Hospital of Shantou University Medical College, Shantou, Guangdong, China; ^3^ Clinical Laboratory, The First Affiliated Hospital of Shantou University Medical College, Shantou, Guangdong, China; ^4^ Radiology department, Cancer Hospital of Shantou University Medical College, Shantou, Guangdong, China

**Keywords:** area under the plasma concentration-time curve, fluorouracil, dihydropyrimidine dehydrogenase, thymidylate synthetase

## Abstract

Fluorouracil (5-FU) has been wildly used as a primary medication in the treatment of solid tumors including colorectal cancer. The treatment efficacy and toxicity of 5-FU varies greatly among individuals, suggesting a need for individualized regimen for cancer patients. The present study analyzed the blood concentration of 5-FU and its therapeutic efficacy and toxicity, evaluated the relationship of AUC (area under the plasma concentration-time curve), and the protein expression of DPD (dihydropyrimidine dehydrogenase) and TS (thymidylate synthetase), and therapeutic efficacy and toxicity. It was found that the AUC of 5-FU was 34.16±14.83mg·h/L in this cohort of study. The immunohistochemical analysis revealed 38.96% and 81.82% positive staining for DPD and TS in colorectal cancer tissues, respectively. We demonstrated that the expression of TS is positively correlated with the expression of DPD. There was a positive correlation between AUC and therapeutic efficacy, and gastrointestinal tract and neural toxicity. The expression of neither DPD nor TS had significant correlations with therapeutic efficacy and toxicity. Based on the blood 5-FU concentration and its relationship with treatment efficacy and toxicity, we determined an optimal therapeutic dosage of 5-FU to be equivalent to an AUC=28.03-38.94mg·h/L. Our study will be helpful in providing an individualized medical regimen for the treatment of colorectal cancer patients.

## INTRODUCTION

Colorectal cancer is a common malignancy in the gastrointestinal tract. Currently there are more than 170,000 patients in China being diagnosed annually with colorectal cancer. The morbidity of colorectal cancer is increasing in recent years because of dietary habit, dietary structure and population aging in China. Fluorouracil (5-FU) is a primary drug used to treat solid tumors and is widely used in the chemotherapy of colorectal cancer. However, 5-FU has a narrow therapeutic dose range and its usage displays significant individual difference that often results in elevated toxicity. The wide individual differences in response to 5-FU treatment suggest that calculating the 5-FU dose by using body surface area is insufficient. In addition, clinical research has not shown a significant correlation between the dose of 5-FU calculated based on body surface area, and clinical response [1], although the pharmacological parameters of 5-FU *in vivo* showed significant correlation [2]. Some studies have demonstrated a significant correlation between the area under the plasma concentration-time curve (AUC) and toxicity of 5-FU [3]. Physicians have shown greatly improved clinical response and reduced toxicity by adjusting the dose of 5-FU in patients with colorectal cancer based on monitoring the blood concentration and AUC assessment [4, 5]. As a result, an individualized medical regimen was proposed to use AUC which demonstrates the best therapeutic dose based on the extent of the patient's illness and individuality.

Previous studies have indicated that certain enzymes play important roles in the pharmacological effect of 5-FU [6, 7] and suggested that treatment regimen should consider these enzymes' activities. *In vivo*, 5-FU inhibits thymidylate synthetase (TS) to block DNA synthesis, and is metabolized by dihydropyrimidine dehydrogenase (DPD) to become inactive metabolites. Levels of TS and DPD are different in different individuals, with DPD levels varied for up to 20-fold in general population [8]. Past research has focused primarily on the role of DPD on 5-FU toxicity, rather than the role of DPD and TS on both the clinical efficacy and toxicity. Whether and how DPD and TS affect 5-FU-mediated toxicity by altering the pharmacological parameters of 5-FU (such as AUC) is poorly understood. In the present study, we assess the blood levels of 5-FU and tumor tissue expression of DPD, TS; and, evaluate the influence of DPD and TS on AUC, and correlate them with clinical response and toxicity of 5-FU. We use these results to determine the best therapeutic dose of 5-FU to help establishing individual medical regimen for treating patients with colorectal cancer. Our results will be useful in increasing the therapeutic efficacy of 5-FU with reduced toxicity, and can enhance the quality of life of colorectal cancer patients.

## RESULTS

### Blood 5-FU level and its AUC

The steady-state concentration (Css) of 5-FU in blood was analyzed using HPLC. As expected, the blood level of 5-FU varied greatly in in these patients. It ranged from 0.33 to 2.26 mg/L, with a mean Css of 0.74±0.32 mg/L. The AUC was evaluated based on: AUC = Css · T (T is the time of constant speed drip) and was found to be 34.16±14.83 mg·h/L, and ranged from 15.06 to 103.73 mg·h/L (Table 1). Using the K-S Test, we found that the Css and AUC level of 5-FU obeyed the normal distribution.

**Table 1:** AUC level in plasma samples of colorectal cancer patients

Mean±SD	Max	Min	95%CI	K-S Test	
				Z	*P*-Value
34.16±14.83	103.73	15.06	30.79~37.53	1.206	0.109

### Expression of TS and DPD in colorectal cancer

**Figure 1:**
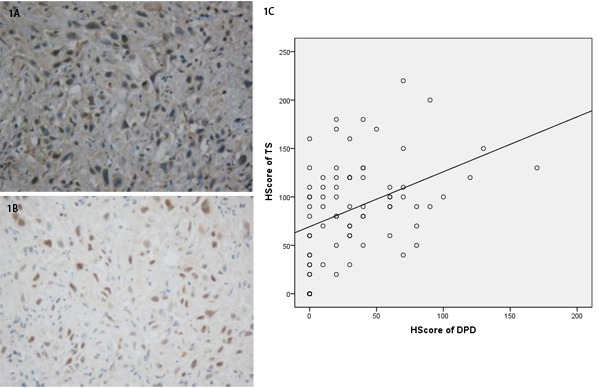
**A.** Immunohistochemical staining of DPD in colorectal cancer tissue. **B.** Immunohistochemical staining of TS in colorectal cancer tissue. **C.** Correlation between TS and DPD level.

The expression of DPD and TS was analyzed using immunohistochemistry and the H-score of staining was obtained. Immunohistochemistry of tumor biopsies revealed positive staining of DPD and TS in colorectal cancer tissues, with a positive expression of 38.96% and 81.82%, respectively. The H-score of the staining and the correlation between the expression of either DPD or TS and AUC was analyzed. There was no significant correlation was found (*P* > 0.05). However, the expression of TS had a significant positive correlation with the expression of DPD (*P* < 0.05,) (Figure 1).

### Relation of AUC and the therapeutic efficacy and toxicity

**Figure 2:**
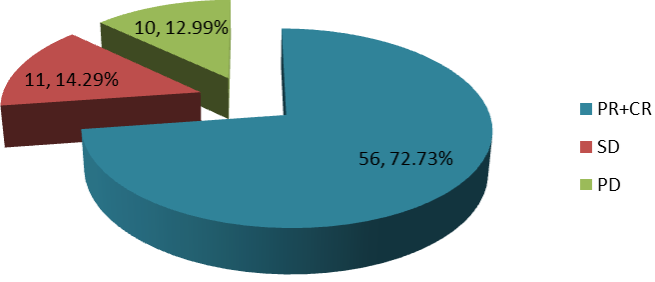
Proportion of therapeutic effect in patients following 5-FU administration CR: complete remission, PR: partial remission, SD: stable disease and PD: progressive disease.

Therapeutic outcome was defined as CR: complete remission, PR: partial remission, SD: stable disease and PD: progressive disease. The therapeutic efficacy (PR+CR/total patients) in this study was 72.73% (Figure 2). The AUC in the PR+CR group was higher than in the SD and PD group (*P* < 0.05, Table 2). These results indicated that the AUC is positively correlated with therapeutic efficacy.

Among the 77 colorectal cancer patients, the occurrence of gastrointestinal tract (GI) toxicity was 92.21% and neural toxicity was 76.62% (Table 3). Patients were divided into high- and low- toxicity groups. Patients in the high toxicity group were those with grade 3-4 in GI and neural toxicity, whereas those with grade 0-2 both in GI and neural toxicity were placed in the low toxicity group. The proportion of high toxicity patients accounted for 35.06%. We found that the AUC of high toxicity group was significantly higher than that in the low toxicity group (Table 4). The AUC was positively correlated with therapeutic efficacy, GI toxicity and neural toxicity (Table 5, *P* < 0.05). The relation of AUC to therapeutic efficacy and toxicity was summarized in Table 5.

**Table 2:** AUC level and therapeutic efficacy

	PR+CR	SD	PD	*P*-Value
AUC(mg·h/L)	38.94±14.52	23.45±5.46	19.17±1.79	<0.01

**Table 3:** 5-FU Toxicity classification in colorectal cancer patients

Classification *n* (%)	Gastrointestinal toxicity 71 (92.21)	Neural toxicity 59 (76.62)
Grade 1-2	46 (59.74)	56 (72.73)
Grade 3-4	25 (32.47)	3 (3.90)

**Table 4:** AUC levels and toxicity

Groups	*n* (%)	AUC (mg·h/L)
Low toxicity	50 (64.94)	28.03±8.82
High toxicity	27 (35.06)	45.51±17.04**

** *P* < 0.01

### Relation of expression of DPD and TS to therapeutic efficacy and toxicity

We found that the expression of neither DPD nor TS correlated with the therapeutic efficacy and toxicity (Table 5). When patients were divided into two groups based on positive or negative DPD and TS immunohistochemical staining, there was no significant difference existed in therapeutic efficacy or toxicity between these two groups. Therefore, the expression of neither DPD nor TS was correlated with the therapeutic index and toxicity.

**Table 5:** Correlation between AUC, DPD, and TS vs. effect and toxicity

		DPD	TS	Therapeutic efficacy	Alimentary canal toxicity	Neuro-virulence
AUC	*r*	-0.167	-0.009	0.493**	0.695**	0.550**
	*P*	0.146	0.936	0.000	0.000	0.000
	*n*	77	76	77	77	77
DPD	*r*		0.420**	0.040	-0.096	-0.183
	*P*		0.000	0.730	0.405	0.111
	*n*		76	77	77	77
TS	*r*			0.181	-0.002	-0.143
	*P*			0.118	0.988	0.217
	*n*			76	76	76
Therapeutic	*r*				0.194	0.414**
efficacy	*P*				0.090	0.000
	*n*				77	77
Alimentary	*r*					0.409**
canal	*P*					0.000
toxicity	*n*					77

** means significant correlation

### Optimal therapeutic dose of 5-FU for colorectal cancer patients

The mean AUC levels for 5-FU was 28.03±8.82mg·h/L in the low toxicity group and 45.51±17.04 mg·h/L in the high toxicity group. We compared the therapeutic efficacy (the occurrence of patients with PR+CR) between patients with an AUC higher *vs*. lower than 28.03mg^.^h/L. Therapeutic efficacy was 94.00% *vs*. 33.33% in the ≥ 28.03 *vs*. < 28.03mg·h/L groups, respectively (*P* < 0.05, Table 6).

We then divided the patients into two groups by therapeutic efficacy. Patients with SD+PD were placed as the poor response group, whereas patients with PR+CR were placed as the high response group. The AUC levels of the poor and high response groups were 21.41±4.60 and 38.94±14.52 mg·h/L, respectively. The mean incidence of high grade toxicity, in patients with an AUC ≥ 38.94*vs*. < 38.94 mg·h/ L, was 70.97% *vs*. 10.87% (*P* < 0.05, Table 7). Based on the above results, the best therapeutic dose of 5-FU was equivalent to an AUC = 28.03-38.94mg·h/L

**Table 6:** Therapeutic efficacy of colorectal cancer patients (divided by 28.03 mg·h/L)

	5-FU AUC (mg·h/L)	
Groups	<28.03	≥28.03
PR+CR	9	47
SD+PD	18	3
Total	27	50
Efficacy (%)	33.33	94.00 **

** *P* <0.01

**Table 7:** Toxicity of colorectal cancer patients (divided by 38.94 mg·h/L)

	5-FU AUC (mg·h/L)	
Groups	<38.94	≥38.94
Low toxicity	41	9
High toxicity	5	22
Total	46	31
High toxicity rate (%)	10.87	70.97 **

** *P* < 0.01

## DISCUSSION

5-FU is widely used in combination with other drugs in the treatment of solid tumors, such as colorectal, gastric and breast cancer. Although there is an increasing use of oxaliplatin and irinotecan, 5-FU remains an important part of the drug combination regimen [9]. The FOLFOX regimen is commonly used in Asia and in our cancer hospital. It was reported that using FOLFOX regimen in colorectal cancer patients, the highest and lowest 5-FU Css can varied as much as 100-fold in different individuals. The variation of 5-FU blood concentration may account for its limited clinical application. We found a significant difference in the 5-FU AUC or Css in colorectal cancer patients in our facility and others who also use FOLFOX regimen [10]. One of the reasons of the difference might come from the 5-FU dosage prescribed by physicians based on the calculation of the body surface area. It could also result from the different metabolism among individual patients.

DPD and TS play important roles in 5-FU biotransformation. This study explored the relationship of AUC and the expression of DPD and TS. It was anticipated that the expression of DPD should negatively correlate with AUC because 5-FU is degraded by DPD. Also, the sensitivity to 5-FU could be indicated by the expression of TS. However, we did not find any significant correlation between AUC and the expression of either DPD or TS. The expression of DPD is 38.96% positive in this cohort of patients which is much lower than the 73.58% reported by Xiao et al. [11] and 93-96% reported by Ezzeldin [12]. DPD gene polymorphism has been proposed as the primary factor for the difference of DPD expression [13]. The expression of TS was 81.82% positive in this study. Ulrich et al. have reported that the TS gene polymorphism on the nucleotide position 28 and 1494 downstream of the transcription start site could influence expression of TS mRNA [14]. The gene polymorphism of both DPD and TS might explain the different expression of these two proteins in different patients population.

Adverse reactions of 5-FU include GI toxicity, myelosuppression, neural toxicity and occasional cardiotoxicity. The CTCAE4.0 standard is used in our cancer center to evaluate clinical efficacy of medications. For toxicity assessment, we focus on the GI toxicity and neural toxicity of 5-FU, since these two toxicities are the primary side effects for 5-FU. Our FOLFOX regimen gives us a 72.73% clinically effective response rate in the patients, which is higher than the 35 and 48% response rate reported by other investigators [10, 15]; and it is also higher than a previous FOLFOX4 study in Korea [16]. Although the incidence of GI toxicity is 92.21%, 59.74% of them were grade 1-2 toxicity. No grade 4 toxicity is observed in this study. The incidence of neural toxicity is 76.62%, with only 3.90% being of grade 3. There is a higher incidence of GI toxicity and lower neural toxicity in this study compared to others. Sugihara reported a 6.3% incidence of diarrhea ( > grade 3) in Asian patients treated with a FOLFOX regimen [17]. There was a report indicated the incidence of neural toxicity of 12.8% for grades 3-4 [18], and 78.9% for grades 1-2. Nausea (grade 3-4) incidence has been reported to be 5.3% in Japanese patients [19]. Caucasians have a higher incidence of adverse reactions than Negros, indicating ethnic differences in sensitivity to 5-FU-mediated toxicity [20]. Our results are in consistent with previous studies that shown positive correlations between clinical efficacy, toxicity and AUC [21, 22]. We demonstrate that the efficacy and toxicity are positively correlated with AUC. Therefore, determining the 5-FU dosage based on AUC is important to optimize individual treatment results.

Individuals with higher levels of DPD display decreased efficacy because of elevated modification of 5-FU. Over expression of DPD mRNA correlates with resistance to 5-FU, while low DPD expression results in better efficacy and higher toxicity [23, 24]. Polymorphisms in the DPD gene are reported to be associated with severe 5-FU toxicity in Japanese [25]. Ezzeldin demonstrated that 50% of patients with grade 3-4 toxicity have a gene mutation or low activity of DPD [12]. There is no significant correlation between the efficacy, toxicity and DPD level [10, 11]. We did not find any significant correlations between either clinical efficacy or toxicity with DPD levels, one of the reasons might be the limited cases in the study cohort. However, we did observe some cases who demonstrated high clinical efficacy and high toxicity with DPD deficiency.

Although a meta-analysis showed high levels of TS is correlated with poorer overall survival [28], the influence of TS on the efficacy and toxicity of 5-FU is still being debated. Some investigators show both positive and negative correlations in the treatment of colorectal cancer patients [26]. We did not find any significant correlations between TS level and either efficacy or toxicity.

It should be noted that all patients enrolled in this study were at the early stage and had the Karnofsky scored more than 60, which meant that this conclusion may only fit for the patients with early stage colorectal cancer.

In summary, the use of 5-FU therapeutic dosage varied greatly in different regions [27]. In the current study, we determine an optimal therapeutic dosage of 28.03-38.94mg·h/L for Chinese colorectal patients. Nevertheless, it is necessary to design individual regimens for colorectal cancer patients by monitoring AUC, instead of BMI. An optimal therapeutic dosage could enhance treatment efficacy and improve patients' quality of life. The expression of DPD and TS might serve as biomarkers for designing treatment regimens, but their roles need to be further explored.

## MATERIALS AND METHODS

### Study subjects

This study was approved by the Human Ethics Committee of Shantou University Medical College (No. 2015030915). Patients received detailed explanations of the study procedure and potential consequences and gave their written informed consent before enrollment.

A total of 77 colorectal cancer patients (53 males and 24 females), age ranges from 36-73 years old (average 56.27), who were in need to be administered with 4-6 courses of chemotherapy with the FOLFOX6 regimen according to doctors' assessment, were recruited in the tumor hospital of Yuedong District in China from January to September 2013. These patients had Karnofsky scores higher than 60 and were predicted a survival time of more than 3 months. The FOLFOX6 regimen contained oxaliplatin (85 mg/m^2^, day1), calcium folinate (0.4 g/m^2^, day1), 5-FU (0.4g/m^2^, day1, intravenous injection) and 5-FU (2.4 g/m^2^, constant speed drip). The therapeutic efficacy and adverse reaction of regimen were evaluated by their attending doctors.

### Sample collection and evaluation

Blood: Five milliliters of venous blood was drawn from each patient in the first course 24 hours after initiating 5-FU intravenous drip. Blood samples were centrifuged at1739g for 10 min, and then stored in a refrigerator until used. Plasma (800 μL) was mixed with 3 mL of ethyl acetate:absolute ethyl alcohol (75:25), vortex mixed for 3 min and centrifuged at 1739g for 10 min. The upper layer was transferred to a clean tube, and then 3 mL of ethyl acetate:absolute ethyl alcohol (75:25) was added to and mixed with the lower layer, vortex mixed for 5 min, and centrifuged as above. The two supernatant fractions were combined, dried under nitrogen, then dissolved in water:methyl alcohol (97:3), filtered and analyzed by high performance liquid chromatography (HPLC-EX1600) to obtain a steady-state concentration(Css) of 5-FU. The conditions for HPLC analysis were: Mobile phase with ethyl acetate:absolute ethyl alcohol (75:25); Injection volume 20 μL; Column temperature 25°C; Flow rate 0.6 ml/min. The recovery of this method was 92.0%. After the Css of 5-FU was determined, the AUC was calculated by AUC = Css · T (T is the time of constant speed drip).

Tissue: tissue samples were collected from the biopsy, fixed in formalin and embedded in paraffin. Paraffin sections were cut, and dried at 60±5°C for 2 hours. Dried samples of the tissue were sent to the Jinyu Medical Examination Center in Guangzhou to assess the protein expression of TS and DPD by immunohistochemistry. Anti-TS antibody (Shanghai Quanhui Company, China) and anti-DPD antibody (Abcam, England) were used as primary antibodies. All stained sections were evaluated by two blinded pathologists. Re-evaluation was performed when the results were different. The criteria of evaluation on the pathological tissue were as follows: one random selection from the high staining pathological sections while five random selections from the low staining ones. Scores were given according to the staining intensity, 0 = negative staining, 1 = faint staining, 2 = medium staining, 3 = strong staining. H score = Σ(I×PC) was used to evaluate the expression of DPD and TS, here “I” presents staining intensity and “PC” presents positive staining percentage. The staining of DPD and TS with H score higher than 30 was considered positive staining.

### Therapeutic efficacy and toxicity assessment

Treatment and response results were routinely recorded in patients' medical charts. Therapeutic efficacy was assessed based on the Response Evaluation Criteria in Solid Tumors 1.0.(WHO standard). Toxicity was evaluated using the Common Terminology Criteria for Adverse Events 4.0 of the U.S. Department of Health and Human. We reviewed the patients' medical records and assessed the therapeutic effect including CR (complete remission), PR (partial remission), SD (stable disease) and PD (progressive disease). Therapeutic efficacy was calculated by the formula (According to the WHO standard): (CR+PR)/total cases ×100%. In this study, doctors evaluated the neural toxicity (including of dysesthesia, paresthesia, peripheral movement disorders, peripheral sensory nerve disorder) and toxicity of the gastrointestinal tract (including of nausea, emesis, diarrhea and constipation) of the patients.

### Statistical analysis

SPSS (Statistical Product and Service Solutions) version 19.0 software was used for statistical analyses. Normality test of data was assessed by the K-S test. Data with a normal distribution was expressed as mean and standard deviation. The differences in the variables were compared using the independent sample *t*-test and ANOVA. Data with a skewed distribution was expressed as median and interquartile range, and differences were compared using the Kruskal-Wallis H and Mann-Whitney U tests. Spearman correlation analysis was performed to analyze the association between DPD and TS, DPD and AUC, TS and AUC, and to analyze how AUC, DPD, and TS correlate with therapeutic efficacy and toxicity.
